# The Blot upon the Brain

**Published:** 1886-10

**Authors:** 


The Blot upon the Brain. *Studies in History and Psychology. By* William W. Ireland, m. d. (*Edin.*) *New York:* G. P. Putnam's Sons. *Chicago:* W. T. Keener. *Octavo, pp. viii, 374. 1886.*

This work is made up of a number of sketches and short articles which have appeared from time to time in the *Journal of Mental Science* and in *Brain.* Most of the historical essays are of great interest, notably those treating of the hallucinations of Mohammed, Luther and Swedenborg; and paper four, on the insanity of power, as illustrated by the Claudian–Julian family, Mohammed Toghlak, and Ivan the Terrible. The value of the book depends upon the value of each essay. There is no connection further than a common binding and an index. The reader will find much of value, that he will look for in vain elsewhere.

